# The safety and feasibility of laparoscopic approach for the management of intrahepatic and extrahepatic bile duct stones in patients with prior biliary tract surgical interventions

**DOI:** 10.1038/s41598-022-18930-1

**Published:** 2022-08-25

**Authors:** Ying-Yu Liu, Tian-Yu Li, Shuo-Dong Wu, Ying Fan

**Affiliations:** Department of General Surgery, Shengjing Hospital of China Medical University, Shenyang, 110004 Liaoning China

**Keywords:** Hepatology, Gastrointestinal diseases

## Abstract

The purpose of this study was to compare the efficacy and safety of laparoscopic and open reoperation for intrahepatic and extrahepatic bile duct stones patients with previous biliary tract surgical procedures. The clinical data were retrospectively analyzed of intrahepatic and extrahepatic bile duct stones patients with previous biliary tract surgical procedures who underwent reoperation in the Second General Surgery Department of China Medical University from January 2012 to February 2018. 44 eligible cases were selected. In accordance with the surgical procedures, they were divided into a laparoscopy group (n = 23) and an open surgery group (n = 21). No statistically significant differences were found in the preoperative general clinical data between the two group. Two patients in the laparoscopy group were converted to open surgery. Comparisons between the two groups showed that the intraoperative blood loss [90.87 ± 62.95 (ml) vs. 152.38 ± 118.82 (ml)], the proportion of postoperative analgesia [10/23 (43.5%) vs. 16/21 (76.2%)], and the length of stay [7.19 ± 5.32 (d) vs. 11.00 ± 4.66 (d)] in the laparoscopy group were significantly lower than those in the open surgery group (*P* < 0.05). Laparoscopic biliary reoperation for intrahepatic and extrahepatic bile duct stones was feasible. Compared with open surgery, laparoscopic surgery has the advantages of less bleeding, a shorter postoperative length of stay, and a lower rate of additional postoperative analgesia.

## Introduction

The causes of intrahepatic and extrahepatic bile duct stones are complex and the recurrence rate is high^1,2^. For patients with recurrence, reoperation is commonly needed. In general, biliary tract reoperation includes endoscopic sphincterotomy (EST), open common bile duct exploration (OCBDE), and laparoscopic common bile duct exploration (LCBDE). Compared with EST, both open and laparoscopic biliary surgery have been favored by doctors and patients because of their ability to preserve the integrity of the Oddi sphincter and their higher stone clearance rate and lower incidence of complications^3–8^.

The history of previous biliary tract surgery increases the difficulty of biliary reoperation and the incidence of surgical complications. It is accompanied by different degrees of severity of tissue adhesion in the abdominal cavity of the patients. Under general conditions, the more extensive adhesion leads to greater the difficulties in surgery. Moreover, a lower security coefficient results in a higher incidence of complications^9^. Thus, the OCBDE procedure was always used for biliary reoperation in the past. Laparoscopic surgery has been increasingly favored by patients and doctors due to the advantages, such as lower trauma severity, rapid recovery, and low postoperative pain, etc. With the development and maturity of technology, the history of previous abdominal surgery is no longer an absolute contraindication for laparoscopic biliary reoperation, but its clinical application is still controversial^9–11^. In this study, to conduct retrospective analysis, we collected the clinical data from 44 patients who underwent biliary tract reoperation in the General Surgery Department of China Medical University from January 2012 to February 2018. The main purpose of the study was to explore the feasibility of laparoscopic reoperation for intrahepatic and extrahepatic bile duct stones.

## Material and methods

### Patient

From January 2012 to February 2018, patients with intrahepatic and extrahepatic bile duct stones who received biliary tract reoperation at the General Surgery department of China Medical University Shengjing Hospital were collected. The previous biliary procedures of patients include cholecystectomy or bile duct exploration, whether laparoscopy or laparotomy. The diagnosis of bile duct stones was based on imaging examination. Patients with bile duct stenosis, bile duct malformation, liver cirrhosis and suspicious malignant tumors of the biliary system were excluded. A total of 44 patients were included in the study. The surgical procedure was determined according to the location of calculi and liver function reserve. According to the surgical procedures performed, the patients were divided into two groups: laparoscopy surgery group (n = 23) and open surgery group (n = 21). The types of previous biliary tract surgery are summarized in Table 1. The general characters of patients in the two groups are shown in Table 2. The follow-up date was June 2022. Follow-up consist of telephone and outpatient follow-up.

**Table 1:** The types of previous surgery.

Type of previous upper abdominal surgery	N (%)	
	Laparoscopy group	Open group
**Once time**		
OC	9 (39.1)	4 (19.0)
Cholecystectomy with stone retrieval	2 (8.7)	0 (0)
LC	1 (4.3)	1 (4.8)
OCBDE	7 (30.4)	12 (57.1)
LCBDE	2 (4.7)	2 (9.5)
**More time**		
OC + LLH	1 (4.3)	0 (0)
OC + radical resection of sigmoid colon cancer	1 (4.3)	0 (0)
LC + LLH	0 (0)	1 (4.8)
OC + OA + distal subtotal gastrectomy	0 (0)	1 (4.8)

*LC* laparoscopic cholecystectomy, *OC* open cholecystectomy, *OCBDE* open common bile duct exploration, *LCBDE* laparoscopic common bile duct exploration, *LLH* left lateral sectionectomy.

**Table 2:** Basic patient data.

	Laparoscopy group	Open group	*P*-value
**Gender**			0.555
Male (n, %)	10 (43.4)	11 (52.4)	
Female (n, %)	12 (56.6)	10 (47.6)	
Age (years)	66.5 ± 8.8	64.0 ± 12.4	0.462
Previous surgical history	–*	–*	0.173
Diameter of common bile duct(mm)	14.65 ± 4.79	14.00 ± 5.70	0.682
Bilateral IHD stones (n, %)	2 (8.7)	4 (19.0)	0.318
White blood cell count(× 10^9^/L)	6.43 ± 3.81	6.36 ± 3.34	0.945
ALT (U/L)	96.83 ± 84.60	90.43 ± 72.07	0.789
AST (U/L)	63.83 ± 28.16	55.86 ± 37.48	0.538
Total bilirubin(umol/L)	38.44 ± 15.49	47.59 ± 16.36	0.744
Direct bilirubin(umol/L)	36.03 ± 8.64	35.66 ± 18.36	0.932

*ALT* alanine aminotransferase, *AST* aspartate aminotransferase, *IHD* intrahepatic bile duct.

*See Table 1 for details.

### Laparoscopy technique

After successful general anesthesia, the patient was placed in a supine position. Pneumoperitoneum was achieved by Hasson technique or Veress technique through the subumbilical incision. When subumbilical adhesions occur, blunt finger dissection is required. The pressure was maintained at 12–14 mmHg. A 10 mm trocar was selected as the observational port to place into the laparoscope for observation. Then, change the patient’s position to 15° to 20° reverse Trendelenbrug’ s position. Another three trocar were established under laparoscopic guidance. A 10 mm trocar was inserted below the xiphoid as the main working port. Two 5 mm trocar were inserted right costal margin as the auxiliary working port.

Because all the patients had a history of cholecystectomy or biliary tract exploration, most of the patients had adhesion between right upper abdominal wall and omentum, colon and lower liver margin, stomach wall and hepatoduodenal ligament to varying degrees under laparoscopy. The surgeon separated the adhesion around the operation area until the hepatoduodenal ligament was revealed. For adhesion of different natures, ultrasonic scalpels, and other equipment should was flexibly selected for treatment of the adhesions. Unnecessary dissection is infeasible. After confirmation of the position of the common bile duct on the hepatoduodenal ligament, which was separated by a fine puncture needle, the common bile duct incision was performed. The stone was taken by squeezing the biliary tract, flushing with a flushing pipe, choledochoscopy combined with the mesh basket, crushing equipment, etc. After the biliary exploration, indwelling T-tube drainage was necessary. For patients with complete removal of stones, the integrity of papillary muscle function, dilated biliary tract, and no obvious inflammation, primary suture was selected. All patients were placed with a peritoneal drainage tube.

### Open technique

The technique of subtotal cholecystectomy was similar to laparoscopic approach. After successful general anesthesia, the patient was placed in a supine position. The original surgical incision approach was selected and the original incision scar was removed. When the position of the common bile duct was clearly exposed under the field of view, a fine puncture needle was used for puncture. The choledochotomy and exploration were performed, and the stones were out after the bile flowed out. The procedure of stone removal is the same as laparoscopy. According to criteria identical to those used in laparoscopic surgery, primary suture or indwelling T-tube drainage was selected, and all patients were placed with a peritoneal drainage tube.

### Postoperative treatment

After surgery, symptomatic treatments were given according to the conditions of the patients. The stomach tube and urinary catheter were withdrawn in all patients on the first day after surgery, and the patients were fed with fluids. The abdominal drainage tube was withdrawn when the amount of the drainage was less than 10 ml/d. The patients with indwelling T-tube were informed to gradually close the T-tube and were then advised to completely close the T-tube. Choledochoscopy were performed two weeks postoperatively. Patients with residual stones underwent choledochoscopic lithotomy, and the T-tube was removed after confirming that no residual stones had remained in the bile duct.

### Retrospective analysis

The general conditions (gender, age, previous surgical history, common bile duct diameter, and laboratory indicators), intraoperative conditions (operation time and intraoperative blood loss), and postoperative conditions (postoperative exhaust time, postoperative length of stay, hospitalization costs, postoperative complications and long-term recurrence) were compared between the two groups of patients. All methods are carried out in accordance with relevant guidelines and regulations and approved by Research Ethics Committee of Shengjing Hospital of China Medical University. The need of informed consent was waived by the Research Ethics Committee of Shengjing Hospital of China Medical University.

### Statistical analysis

Statistical analysis was performed on the data using SPSS 23.0 statistical software. The measurement data are expressed as mean ± standard deviation ($\overline{X }$ ± S). The t-test was used for comparisons between the two groups. The count data was expressed as a rate (%), and the chi-square test was utilized for comparisons between the two groups. The difference was statistically significant at *P* < 0.05.

## Result

### Perioperative outcomes

The perioperative outcomes of two groups are listed in Table 3. Two patients with extensive abdominal adhesion in laparoscopy group were converted to open surgery. The conversion rate was 8.7%. Three patients in laparoscopy group and two patients in open group underwent primary suture of biliary tract. No significant differences were found in the operation time, postoperative exhaust time, and total hospitalization costs between the two groups (*P* > 0.05). Laparoscopy group exhibited lower intraoperative blood loss (90.87 ± 63.95 vs. 152.38 ± 118.82, *P* = 0.036) and postoperative length of stay (7.19 ± 5.32 vs. 11.00 ± 4.66, *P* = 0.048) compared to open group.

**Table 3:** Comparison of surgical-related indicators between the two groups.

	Laparoscopy group	Open group	*P*-value
Operation time (min)	153.83 ± 56.97	165.76 ± 52.39	0.475
Intraoperative blood loss (ml)	90.87 ± 62.95	152.38 ± 118.82	0.036
Postoperative exhaust time (d)	3.43 ± 0.79	3.19 ± 1.40	0.475
Postoperative length of stay (d)	7.19 ± 5.32	11.00 ± 4.66	0.048
Hospitalization costs (thousand Yuan)	41.60 ± 14.78	41.11 ± 15.25	0.914

### Postoperative analgesia and complications

The postoperative analgesia and complications are listed in Table 4. All patients had no biliary stricture. The proportion of postoperative additional analgesia in laparoscopy group was lower than that in open group, the difference was statistically significant (43.5% vs. 76.2%, *P* = 0.027). Meanwhile, there was no significant difference in the incision infection rate the occurrence rate of bile leakage, and the residual stones between the two groups (*P* > 0.05). The patients with residual stones in both groups were bilateral intrahepatic bile duct stones. Follow-up was conducted in June 2022. There were 2 patients in the laparoscopic group and 3 patients in the open group were lost to follow-up, with a follow-up rate of 88.9%. A total of 8 patients in the laparoscopic group (38.1%) and 5 patients in the open group (27.8%) had stone recurrence (*P* < 0.05). The average recurrence rate was 33.3%.

**Table 4:** Comparison of postoperative analgesia and complications between the two groups.

	Laparoscopy group	Open group	*P*-value
Postoperative analgesia	10 (43.5%)	16 (76.2%)	0.027
Postoperative bile leakage	1 (4.3%)	1 (4.8%)	1
Incision infection	1 (4.3%)	3 (14.3)	0.535
Residual stone	2 (8.7%)	1 (4.8%)	0.23

## Discussion

Before the widespread application of laparoscopic and endoscopic techniques, OCBDE was the only option for the treatment of recurrent intrahepatic and extrahepatic bile duct stones after biliary tract surgery. In the 1970s, EST gradually replaced the OCBDE in the treatment of recurrent common bile duct stones. However, EST leads to complications, such as acute pancreatitis and reflux cholangitis. Moreover, EST has the inherent disadvantage of destroying the integrity of the Oddi sphincter and incapability to treat intrahepatic bile duct stones^12^. Hence, biliary tract reoperation is still widely used. The choice between open and laparoscopic surgery for the second operation in patients with a previous surgery history is still controversial. In the past, previous abdominal surgery history was considered a contraindication to laparoscopic reoperation for hepatobiliary and pancreatic resection, as it could increase the risk of bleeding and intestinal and biliary tract injuries^10^. In recent years, with the continuous development of laparoscopic techniques, the previous history of abdominal surgery is no longer an absolute contraindication for laparoscopic reoperation. Many studies have confirmed the safety and feasibility of laparoscopic reoperation^9,10,13^. However, few reports exist on re-performing the LCBDE in patients with a history of biliary surgery. This study retrospectively compared the clinical data of laparoscopic and open reoperation for intrahepatic and extrahepatic bile duct stones to explore the feasibility of laparoscopic biliary tract reoperation.

During laparoscopic reoperation, the first trocar position had to be chosen away from the original surgical incision. Generally, the peri-umbilicus position was selected, and the pneumoperitoneum was established by open technique. After the abdominal cavity was opened, under the laparoscopic direct vision, 1–2 trocars were established at a position with relatively less adhesion, which were below the xiphoid process, under the costal margin of the midclavicular line, and under the costal margin of the right anterior axillary line. Then, the omentum and the intestine were separated with an ultrasonic scalpel or tissue scissors near the abdominal was until all the cannulas were placed in.

Finding the common bile duct was the critical step of the surgery. Under normal circumstances, the adhesion of the original surgical area is concentrated mainly in the hilar part, and the adhesion is the most commonly observed in the omentum, colonic hepatic curvature, gastric antrum, and the duodenal bulb. In the separation of adhesions, the safety is to be carefully confirmed, and then the combination of blunt and sharp separation should be used. After loosening the adhesion tissues and resolving the boundary of adhesion, the hard and dense adhesion was carefully separated. From experience, for different types of adhesion, different tools should be flexibly used for careful separation. Importantly, on the premise of meeting surgical requirements, excessive release of adhesions should not be done. Otherwise, the probability of secondary damage increases. Moreover, cavity organs must be protected. When it is determined that the colon, stomach or duodenum are closely adhered to the liver and cannot be separated, part of the liver parenchyma can be sacrificed and separated with the adhesions. The liver margin, the cystic duct residual, the non-absorbable clip, and the surgical suture in the last surgery were all anatomical landmarks for finding the common bile duct. For patients with clear common bile duct, direct incisions could be performed for stone removal. If it was difficult to look for the common bile duct, the puncture needle should be used for puncture according to the above-mentioned marks. If there was bile overflow, the position of the common bile duct can be confirmed.

After the biliary exploration, it is generally necessary to indwell T tube drainage. For patients with complete removal of stones, integrity of papillary muscle function, dilated biliary tract and no obvious inflammation, primary duct closure can be performed^11,14^. In this study, three patients in laparoscopy group, and two patients in open group underwent primary duct closure. No biliary stricture occurred. The biliary stricture was commonly observed in the primary duct closure, which was due to the selection of cases with no obvious bile duct dilatation or excessive suture^15^.

In this study, although the laparoscopic surgery was relatively complicated, the operation time in two groups was not significantly different. This was mainly due to the surgeon’s rich experience in laparoscopy surgery and the saved time from opening and closing the abdomen. The intraoperative blood loss and postoperative length of stay in laparoscopy group were lower than those in open group (*P* < 0.05). This result was related to the good visual field with laparoscopy that reduced the damage to the tissue and the bleeding amount on the wound surface. And the postoperative recovery was rapid. In the long-term follow-up, the follow-up years ranged from 5 to 10 years, the average recurrence rate was 33.3%. In this study, neither of the two surgical procedures showed a superior advantage for stone recurrence.

The hospitalization costs were higher in laparoscopy group, but the difference was not statistically significant (*P* > 0.05). Although laparoscopic surgery required additional laparoscopic equipment, the postoperative length of stay was short and patients recovered quickly. Thus, there was no significant difference in the hospitalization costs. In this study, the postoperative exhaust time in laparoscopy group was slightly longer than that in open surgery group, which was probably due to the small sample size and did not reflect its advantages^16,17^.

During the laparoscopic biliary tract reoperation, the patients with conditions that could not be treated under laparoscopy, such as unstoppable operation area bleeding or suspicious malignant changes, had to be timely transferred to open surgery. In this study, two patients in laparoscopy group had severe intestinal adhesion and were converted to open surgery, after which no complications occurred. The number of cases of postoperative analgesia in laparoscopy group was lower than that in open surgery group (*P* < 0.05). The incidences of bile leakage, biliary stricture, and residual stone rate were not significantly different between the groups (*P* > 0.05). It is worth noting that the residual stones all occurred in patients with bilateral intrahepatic bile duct stones. Compared with laparoscopy, open surgery can better manage bilateral intrahepatic bile duct stones with success rate of 75%.

Laparoscopic surgery ensured a small incision and a good visual field. Therefore, due to its higher accuracy than that of open surgery, the use of this approach successfully minimized the damage to the operation area, reduced the stimulation of the tissues around the operation area, maintained its original anatomy, and greatly diminished the postoperative pain in the patients. There were no biliary strictures in two groups, and the rates of bile leakage and residual stones were not significantly different (*P* > 0.05). Thus, we can conclude that the laparoscopic biliary tract reoperation performed in our investigation was safe.

## Conclusions

In conclusion, this study demonstrated that laparoscopic biliary reoperation is a feasible option for the treatment of intrahepatic and extrahepatic bile duct stones when performed by experienced laparoscopic surgeons. Compared with open surgery, laparoscopic surgery has the advantages of less bleeding, a shorter postoperative length of hospital stays, and a lower rate of postoperative analgesia. These findings are worthy of further generalization and application in clinical practice. This study was a retrospective analysis with a small sample size, which might have caused bias in the selection of samples. Therefore, our positive results require confirmation by clinical prospective studies with large sample sizes.

## Data Availability

The data that support the findings of this study are available from Research Ethics Committee of Shengjing Hospital of China Medical University. But restrictions apply to the availability of these data, which were used under license for the current study, and so are not publicly available. Data are however available from the authors upon reasonable request and with permission of Research Ethics Committee of Shengjing Hospital of China Medical University. The Data are however available from the Prof. Ying Fan upon reasonable request and with permission of Research Ethics Committee of Shengjing Hospital of China Medical University.
